# Gadd45b mediates depressive-like role through DNA demethylation

**DOI:** 10.1038/s41598-019-40844-8

**Published:** 2019-03-15

**Authors:** Benoit Labonté, Yun Ha Jeong, Eric Parise, Orna Issler, Mena Fatma, Olivia Engmann, Kyung-Ah Cho, Rachael Neve, Eric J. Nestler, Ja Wook Koo

**Affiliations:** 1 CERVO Brain Research Centre, Department of Neuroscience and Psychiatry, Faculty of Medicine, Laval University, Québec, Canada; 2 Department of Neural Development and Disease, Korea Brain Research Institute (KBRI), 61, Cheomdan-ro, Dong-gu, Daegu, 41068 Korea; 3 Fishberg Department of Neuroscience, Friedman Brain Institute, Icahn School of Medicine at Mount Sinai, New York, NY 10029 USA; 4 Viral Gene Transfer Core, McGovern Institute for Brain Research, MIT, Cambridge, MA 02139 USA; 5 Present Address: Department of Molecular Medicine, Institute of Biotechnology, The University of Texas Health Science Center at San Antonio, San Antonio, TX 78229 USA

## Abstract

Animal studies using chronic social defeat stress (CSDS) in mice showed that brain-derived neurotrophic factor (BDNF) signaling in the mesolimbic dopamine (DA) circuit is important for the development of social aversion. However, the downstream molecular targets after BDNF release from ventral tegmental area (VTA) DA terminals are unknown. Here, we show that depressive-like behaviors induced by CSDS are mediated in part by Gadd45b downstream of BDNF signaling in the nucleus accumbens (NAc). We show that *Gadd45b* mRNA levels are increased in susceptible but not resilient mice. Intra-NAc infusion of BDNF or optical stimulation of VTA DA terminals in NAc enhanced *Gadd45b* expression levels in the NAc. Importantly, *Gadd45b* downregulation reversed social avoidance in susceptible mice. Together, these data suggest that Gadd45b in NAc contributes to susceptibility to social stress. In addition, we investigated the function of Gadd45b in demethylating CpG islands of representative gene targets, which have been associated with a depressive phenotype in humans and animal models. We found that *Gadd45b* downregulation changes DNA methylation levels in a phenotype-, gene-, and locus-specific fashion. Together, these results highlight the contribution of Gadd45b and changes in DNA methylation in mediating the effects of social stress in the mesolimbic DA circuit.

## Introduction

Animal studies using chronic social defeat stress (CSDS) in mice, an ethologically validated model of aspects of depression in mice^1,2^, previously showed that the mesolimbic dopamine (DA) circuit is critically involved in the development of social aversion and other behavioral abnormalities^3,4^. Indeed, CSDS in mice increases the activity of dopamine (DA) neurons in the ventral tegmental area (VTA) that project to the nucleus accumbens (NAc)^3,5^. Furthermore, optogenetic stimulation of this VTA to NAc pathway increases susceptibility to CSDS via a mechanism involving release of brain-derived neurotrophic factor (BDNF) from VTA DA neuron terminals rather than dopaminergic signaling^4^. BDNF signaling in NAc promotes stress susceptibility through its tyrosine kinase receptor, TrkB, however, the molecular mechanisms underlying these effects remain unknown.

Growing evidence implicates transcriptional alterations induced by CSDS in several limbic brain regions including the NAc in stress susceptibility^6^, and these alterations in stressed mice have been paralleled by similar transcriptional investigations in the post-mortem brains of patients with major depression^7^. While the molecular mechanisms underlying these transcriptional changes are a matter of intense investigation, recent findings suggest a causal link between epigenetic mechanisms, including DNA methylation, histone modifications, and chromatin remodeling, and changes in gene expression (reviewed in^8,9^). Indeed, besides a global reorganization of chromatin complexes, changes in DNA methylation and hydroxymethylation in the NAc have been associated with the effects of CSDS^10–12^. Similarly, genome-wide assessments of DNA methylation changes in human brain previously revealed global reorganization of DNA methylation profiles, associated with psychiatric disorders including major depression, psychosis, bipolar disorder, post-traumatic stress disorder (PTSD), and child abuse^13–17^.

*Gadd45b*, a member of the growth arrest and DNA damage (Gadd45) gene family, is a DNA demethylating candidate. In brain, Gadd45b has been shown to be critically involved in activity-induced neurogenesis by decreasing site-specific DNA methylation in the regulatory regions of the *Bdnf* and fibroblast growth factor 1 (*Fgf1*) genes^18^. Subsequent reports showed that, by regulating DNA methylation levels at precise gene loci, Gadd45b regulates juvenile behavior and pro-inflammatory cytokine production^19^, while influencing long-term memory formation and synaptic plasticity^20,21^. Furthermore, analyses of human post-mortem tissue revealed increased *Gadd45b* mRNA and protein expression in the parietal cortex of psychotic patients^22^. Together, these findings raise the possibility that, by changing DNA methylation levels at specific gene loci, Gadd45b might modulate the molecular cascades regulating stress susceptibility.

In the present study, we assessed the involvement of Gadd45b in mediating the molecular and behavioral effects of CSDS. Our findings suggest that alteration of *Gadd45b* expression in the NAc, downstream of BDNF signaling, is involved in mediating the stress susceptibility in mice by interfering with the establishment of DNA methylation patterns at specific gene loci in this brain region.

## Results

**Figure 1:**
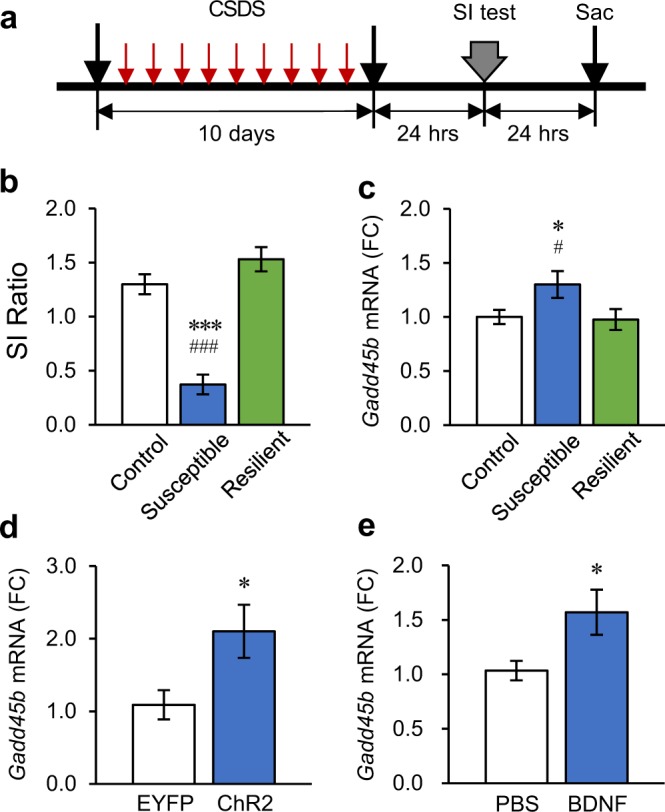
Chronic social defeat stress (CSDS) induces *Gadd45b* in the nucleus accumbens (NAc) of susceptible mice. (**a**) Schematic diagram depicting the experimental procedure for CSDS. (**b**) Repeated CSDS induces social avoidance in susceptible but not resilient mice [One-way analysis of variance (ANOVA), *F*_(2,27)_ = 38.22, *p* < 0.001, Control *n* = 10, Susceptible *n* = 10, Resilient *n* = 10]. Social avoidance was expressed as social interaction ratio (SI ratio) calculated by time spent in the interaction zone with a social target/time in the interaction zone without a social target. **c**, CSDS induces *Gadd45b* mRNA levels in NAc of susceptible but not resilient mice (*F*_(2,27)_ = 3.365, *p* < 0.05, Control *n* = 10, Susceptible *n* = 10, Resilient *n* = 10). mRNA expression levels were expressed as fold change (FC) compared to control group. (**d,e**) Phasic stimulation of the VTA-NAc DA pathway (**d**, Student’s *t*-test, *t*_(15)_ = 2.405, *p < 0.05, EYFP *n* = 8, ChR2 *n* = 9), or intra-NAc BDNF infusion (**e**, Student’s *t*-test, *t*_(17)_ = 2.281, *p < 0.05, PBS *n* = 9, BDNF *n* = 10), induces *Gadd45b* expression in the NAc. *p < 0.05, ***p < 0.001. Bar graphs show mean ± SEM.

*Gadd45b* is an activity-induced immediate early gene in mature hippocampal neurons^18^. As chronic social stress is known to alter transcriptional profiles in several brain regions including the NAc^23^, we first tested whether *Gadd45b* expression is altered following chronic social stress. For this experiments, c57bl/6 mice were subjected to social defeat stress for 10 days and then assessed for social interaction with a social target (Fig. 1a). Ten days of CSDS induced a strong social avoidance phenotype (Fig. 1b, Supplementary Fig. 1a,b) in the susceptible versus resilient and control mice. Our results show that *Gadd45b* expression levels were significantly increased in the NAc of susceptible mice compared to control (Fig. 1c). Importantly, this effect is specific to susceptibility as we found no significant change in *Gadd45b* expression in the NAc of resilient mice. Interestingly, this is in accordance with previous findings showing the involvement of *Gadd45b* in hippocampus in fear conditioning and memory consolidation in mice^21^ and in parietal cortex of humans with psychosis^22^, thus expanding the involvement of *Gadd45b* in NAc in the context of chronic social stress.

Previous data showed that the phasic optogenetic activation of the VTA to NAc pathway in mice—stimulation either of VTA DA neuron cells bodies or of their nerve terminals in NAc—increases susceptibility to social stress^3–5^. We found that the phasic optogenetic activation of VTA terminals in NAc significantly increased *Gadd45b* expression in the NAc (Fig. 1d). Furthermore, the effects of phasic activation of this mesolimbic circuit on susceptibility have been shown to be mediated via the release of BDNF, not DA, from VTA projections in the NAc^4^. Thus, we tested whether the elevated *Gadd45b* expression found in the NAc of susceptible mice is mediated via similar mechanisms. Indeed, intra-NAc infusion of BDNF significantly increased *Gadd45b* expression (Fig. 1e). Together, these data suggest that *Gadd45b* in the NAc is involved in the expression of stress susceptibility through the activation of the mesolimbic circuit and release of BDNF.

**Figure 2:**
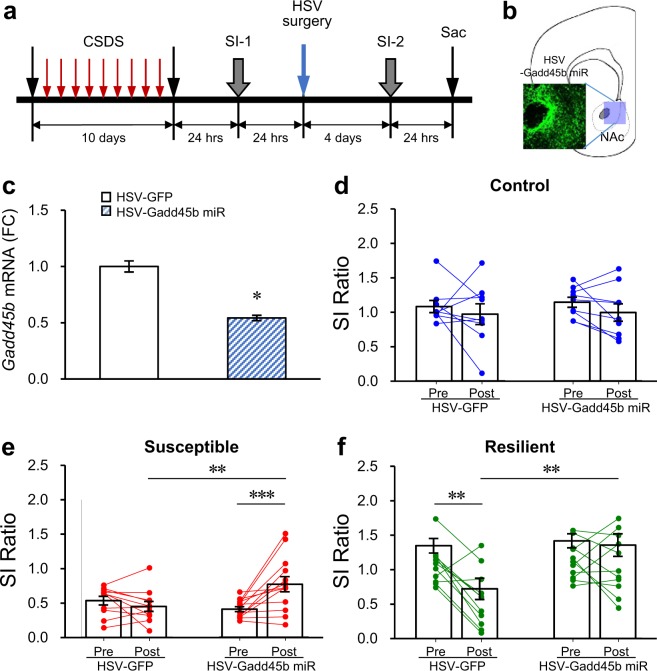
Behavioral effects of local deletion of *Gadd45b* in NAc on social avoidance behaviors. (**a**) Schematic diagram depicting the experimental procedures for CSDS and intra-NAc infusion of HSV-Gadd45b miR. (**b**) Immunohistochemistry after HSV-Gadd45b miR infusion into NAc. (**c**) HSV-Gadd45b miR infused into NAc significantly decreases *Gadd45b* mRNA levels in NAc (*t*_(15)_ = 2.405, *p < 0.05, HSV-GFP *n* = 8, HSV-Gadd45b miR *n* = 9). (**d–f)** Local *Gadd45b* KD in the NAc had no effect on social interaction in stress naïve animals [**d**, Mixed model two-way ANOVA, time effect: *F*_(1,32)_ = 1.305, *p* = 0.262; genetic effect: *F*_(1,32)_ = 0.142, *p* = 0.709; time × genetic effect: *F*_(1,32)_ = 0.028, *p* = 0.868, HSV-GFP (pre- and post-) *n* = 9, HSV-Gadd45b miR (pre- and post-) *n* = 9]. In contrast, local *Gadd45b* KD in this brain region reverses the CSDS-induced social avoidance behavior in susceptible mice [**e**, time effect: *F*_(1,44)_ = 3.25, *p* = 0.078; genetic effect: *F*_(1,44)_ = 1.669, *p* = 0.203; time × genetic effect: *F*_(1,44)_ = 8.398, *p* < 0.01, HSV-GFP (pre- and post-) *n* = 11, HSV-Gadd45b miR (pre- and post-) *n* = 13]. After HSV-GFP infusion into NAc, resilient mice showed reduction of social interaction, whereas intra-NAc infusion of HSV-Gadd45b miR blocks this effect [**f**, time effect: *F*_(1,40)_ = 6.534, *p* < 0.05; genetic effect: *F*_(1,40)_ = 6.913, *p* < 0.05; time × genetic effect: *F*_(1,40)_ = 4.411, *p* < 0.05, HSV-GFP (pre- and post-) *n* = 11, HSV-Gadd45b miR (pre- and post-) *n* = 11]. Mixed model two-way ANOVA with Fisher’s LSD *post-hoc* tests, *p < 0.05, **p < 0.01, ***p < 0.001. Bar graphs show mean ± SEM.

We next tested the hypothesis that reducing *Gadd45b* in the NAc would rescue the social avoidance phenotype induced by 10 days of CSDS in mice using a viral-mediated gene transfer approach (Fig. 2a). We created a viral vector expressing a miRNA that reduces only *Gadd45b* but not *Gadd45a* and *Gadd45r* expression in the NAc (Fig. 2c, Supplementary Fig. 2, see Methods Section). We carried out CSDS on a cohort of mice and identified susceptible and resilient individuals. Twenty-four hrs after the assessment of social interaction (SI-1), we performed intra-NAc infusion of HSV-*Gadd45b* miR or HSV-GFP in control, susceptible, and resilient mice (Fig. 2b) and then reassessed social interaction 4 days post-surgeries (SI-2), a period coinciding with peak expression of HSV vectors (Fig. 2a). Our analysis showed that the viral downregulation of *Gadd45b* expression in the NAc did not affect social interaction in control mice (Fig. 2d). However, in susceptible mice, *Gadd45b* KD (HSV-*Gadd45b* miR) significantly reversed the social avoidance phenotype initially expressed before surgery (Fig. 2e). *Post-hoc* analyses showed that susceptible mice treated with HSV-*Gadd45b* miR spent more time in the interaction zone in the presence of a social target compared to their pre-surgery levels and to susceptible mice treated with HSV-GFP. In resilient mice, HSV-GFP treatment decreased social interaction scores. However, *Gadd45b* KD (HSV-*Gadd45b* miR) in the resilient group successfully normalized social interaction behavior to control conditions (Fig. 2f). We re-analyzed social interaction changes at pre- (SI-1) and post-surgery (SI-2) with social interaction time in each group. We observed the same patterns in the time spent in interaction zone in the presence of a target as in the social interaction ratio (Supplementary Fig. 3). Taken together, these findings suggest that *Gadd45b* in the NAc has a pro-depressant effect on social behaviors following chronic social defeat stress.

The behavioral effects of *Gadd45b* have been suggested to be mediated via its DNA demethylating properties^19–21^. In our results (Fig. 2e), Gadd45b induced-depressive phenotype effect was only shown in susceptible group. Since Gadd45b can modulate the molecular cascades regulating stress susceptibility by changing DNA methylation levels at specific gene loci^18,19^,we tested whether the reversal of CSDS-induced susceptibility by *Gadd45b* KD associates with changes in DNA methylation in a phenotype-, gene-, and locus-specific way in the NAc. We focused our investigation on the *Gad1* (glutamate decarboxylase-1), *Dlx5* (distal-less homeobox 5), *Nrtn* (neurturin), and *Ntrk2* (TrkB) genes (Table 1), each of which has been shown previously to be associated with the effects of stress in both rodents and humans^6,10,12,24^.

**Table 1:** Selected genes for MassARRAY EpiTYPER assay.

Target gene	Region	CpG
*Gad1*	chr2:70562278- 70562531	23
*Dlx5*	chr6: 6881849-6881515	38
*Ntrn*	chr17:56757630-56757402	22
*Ntrk2*	chr13:58806389-58806757	34

**Figure 3:**
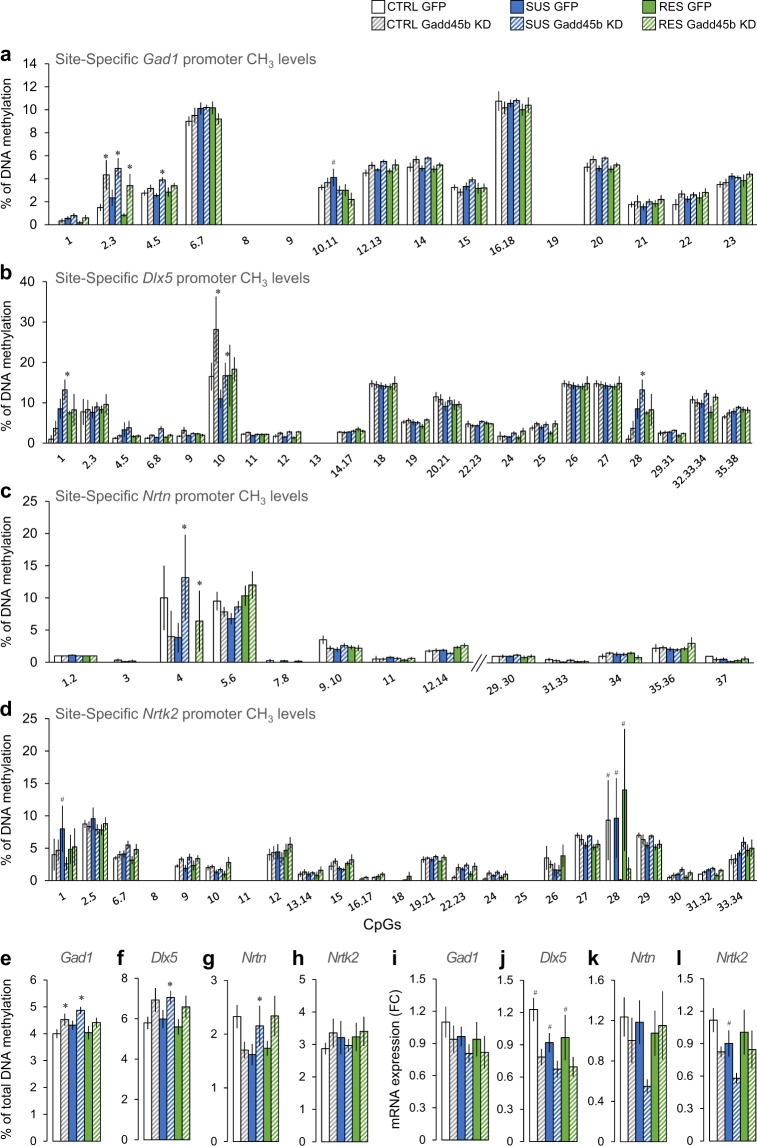
Effects of CSDS and viral-mediated downregulation of *Gadd45b* on DNA methylation and on gene expression of stress-related genes in NAc. (**a,e**) DNA methylation at individual CpGs (**a**) and total DNA methylation (**e**) in the *Gad1* gene promoter in the NAc of control (CTRL; white), susceptible (SUS; blue), and resilient (RES; green) mice with (dashed lines) and without (full box) *Gadd45b* viral KD [phenotype (*F*_(2,137)_ = 4.996, *p* < 0.01), viral KD (*F*_(1,137)_ = 17.648, *p* < 0.001), CpG (*F*_(12,258)_ = 263.746, *p* < 0.001), viral KD by CpG interaction (*F*_(12,258)_ = 4.320, *p* < 0.001)]. (**i)** *Gad1* relative expression in NAc of CTRL, SUS, and RES mice with and without *Gadd45b* KD (*F*_(1,34)_ = 2.101, *p* = 0.078). (**b,f**) DNA methylation at individual CpGs (**b**) and total DNA methylation (**f**) in the *Dlx5* gene promoter in CTRL, SUS, and RES mice with and without *Gadd45b* KD [viral KD (*F*_(1,237)_ = 15.421, *p* < 0.001), CpG site (*F*_(20,414)_ = 74.633, *p* < 0.001), phenotype by CpG site interaction (*F*_(40,410)_ = 2.378, *p* < 0.001)]. (**j)** *Dlx5* relative expression in NAc of CTRL, SUS, and RES mice with and without *Gadd45b* KD (*F*_(1,33)_ = 11.26, *p* < 0.005). (**c,g**) DNA methylation at individual CpGs (**c**) and total DNA methylation (**g**) in the *Nrtn* gene promoter in CTRL, SUS, and RES mice with and without *Gadd45b* KD [CpG site (*F*_(12,268)_ = 37.918, *p* < 0.001), phenotype by viral KD (*F*_(24,268)_ = 1.338, *p* < 0.05)]. (**k)** *Nrtn* relative expression in NAc of CTRL, SUS, and RES mice with and without *Gadd45b* KD (*F*_(1,33)_ = 2.359, *p* = 0.067). (**d,h**) DNA methylation at individual CpGs (**d**) and total DNA methylation (**h**) in the *Ntrk2* gene promoter in CTRL, SUS, and RES mice with and without *Gadd45b* KD [CpG (*F*_(19,352)_ = 17.453, *p* < 0.001) and viral KD by CpG interaction (*F*_(19,352)_ = 4.313, *p* < 0.001]. (**l)** *Ntrk2* relative expression in NAc of CTRL, SUS, and RES mice with and without *Gadd45b* KD (*F*_(1,33)_ = 6.337, *p* = 0.017). For (**a–h**) CTRL GFP *n* = 4, CTRL *Gadd45b* KD *n* = 6, SUS GFP *n* = 9, SUS *Gadd45b* KD *n* = 10, RES GFP *n* = 6, RES *Gadd45b* KD *n* = 5. Mixed model two-way ANOVA with Fisher’s LSD *post-hoc* tests, *p < 0.05, compared to the corresponding GFP group, ^#^*p* < 0.05, compared to the corresponding *Gadd45b* KD group. For (**i**–**l)** CTRL GFP *n* = 5, CTRL *Gadd45b* KD *n* = 6, SUS GFP *n* = 8, SUS *Gadd45b* KD *n* = 10, RES GFP *n* = 6, RES *Gadd45b* KD *n* = 5. Mixed model two-way ANOVA with Fisher’s LSD *post-hoc* tests, ^#^*p* < 0.05, compared to the corresponding *Gadd45b* KD group. Bar graphs show mean ± SEM.

DNA methylation was assessed in the NAc at 23 CpGs within *Gad1’s* gene promoter region (chr2:70562278- 70562531; Fig. 3a,e). Our analysis shows significant main effects of phenotype, viral KD, and CpG with a significant viral KD by CpG interaction effect. *Post-hoc* analysis shows that *Gadd45b* KD significantly increased total DNA methylation levels within the *Gad1* promoter region in control and susceptible mice (Fig. 3e). More interestingly, our *post-hoc* analysis highlighted several CpG sites for which *Gadd45b* KD changed DNA methylation levels (Fig. 3a). Specifically, our data show a significant upregulation of DNA methylation levels at CpG sites 2–3 and 4–5 and a significant downregulation at CpG sites 10–11. Additional sites of differential methylation are shown in Fig. 3a. Our data indicate that these alterations were associated with a trend toward a significant change in *Gad1* expression in the NAc (viral KD main effect; Fig. 3i), although this change was not specific to any particular group.

We found similar effects in the NAc in the promoter region of the *Dlx5* gene for which we covered 38 CpGs (Chr6: 6881849-6881515; Fig. 3b,f). Specifically, our analysis revealed significant viral KD and CpG site main effects with significant phenotype by CpG site interaction. *Post-hoc* analysis shows that *Gadd45b* KD increased significantly total DNA methylation in susceptible mice compared to GFP-treated susceptible mice (Fig. 3f). Here again, *post-hoc* analysis highlighted site-specific effects with significantly higher DNA methylation levels at CpG sites 1, 10, and 28 in HSV-*Gadd45b* miR compared to GFP-treated susceptible mice (Fig. 3b). Additional sites of differential methylation are shown in Fig. 3b. Furthermore, our results show that *Gadd45b* KD also significantly reduced *Dlx5* mRNA expression (viral KD main effect) in the NAc of control, susceptible, and resilient mice compared to their GFP counterparts (Fig. 3j).

DNA methylation of the *Nrtn* gene promoter was evaluated on 22 CpGs (chr17:56757630- 56757402; Fig. 3c,g). Our analysis revealed significant CpG site and phenotype by viral KD interaction effects (Fig. 3c). Interestingly, our *post-hoc* analysis showed that *Gadd45b* KD significantly raised total DNA methylation levels in susceptible mice with no effect in control and resilient individuals (Fig. 3g). Similar to *Gad1* and *Dlx5*, these alterations were associated with a trend toward a significant change in *Nrtn* gene expression in the NAc (viral KD main effect, Fig. 3k), although this change was not specific to any particular group.

Finally, we quantified DNA methylation levels in the NAc at 34 CpGs within the *Ntrk2* gene promoter (*Ntrk2*: chr13:58806389-58806757). Our analysis highlighted CpG and viral KD by CpG interaction effects (Fig. 3d,h). In *Ntrk2* gene promoter, our analysis revealed significantly lower DNA methylation levels in HSV-*Gadd45b* miR-treated susceptible mice compared to GFP susceptible mice at CpG sites 1 and 28 (Fig. 3d). Furthermore, our results show that *Gadd45b* KD significantly reduced *Ntrk2* expression in the NAc of susceptible mice compared to GFP susceptible, control, and resilient animals (Fig. 3l).

## Discussion

In the current study, we present results on the molecular mechanism underlying the expression of susceptibility to chronic social stress in mice. Our findings show that CSDS in mice induces the expression of the DNA demethylase, *Gadd45b*. Importantly, both the optogenetic stimulation of the VTA to NAc pathway and the local infusion of BDNF in the NAc, both of which induce stress susceptibility in mice^3–5^, induced the expression of *Gadd45b* in the NAc. Using a viral approach, we showed that downregulation of *Gadd45b* in the NAc reverses the susceptibility phenotype in chronically stressed mice, suggesting that elevated expression of *Gadd45b* is required for the maintenance of stress susceptibility. We showed further that this reversal (from susceptible to resilient) is associated with DNA methylation changes in the regulatory regions of several genes previously implicated in the effect of stress in both mice and humans. Overall, our findings suggest that alteration of *Gadd45b* expression in the NAc, resulting from dysregulation of the VTA to NAc pathway, is involved in mediating the behavioral effects of stress susceptibility in mice by interfering with the establishment of DNA methylation patterns at specific gene loci in the NAc.

CSDS in mice increases the activity of the VTA to NAc DA circuit in susceptible but not resilient mice^3,23,25^. Optical stimulation of dopaminergic projections from the VTA to the NAc induces stress susceptibility, as measured by reduced social interaction and other behavioral abnormalities^3,4^, through a BDNF-dependant signaling mechanism^1,4,5,23^. Indeed, we previously showed that the behavioral impact of VTA-NAc pathway stimulation is blocked by the intra-NAc administration of a Trkb antagonist but not DA receptor antagonists^4^. Together, this suggests that BDNF signaling in the NAc is a downstream mediator of the deleterious effects of sustained activation of VTA to NAc DA neurons induced by chronic stress. Our present results suggest that *Gadd45b* is one downstream molecular target of BDNF. *Gadd45b* expression was significantly upregulated in the NAc of susceptible mice following CSDS, an effect that was reproduced following either: 1) prolonged optical activation of VTA to NAc DA projections or 2) the intra-NAc infusion of BDNF, two procedures known to increase stress susceptibility and to reproduce the circuit alterations induced by CSDS. These findings thus establish *Gadd45b* as a novel downstream molecular target of BDNF in the NAc, which contributes to the behavioral effects of CSDS.

Previous studies highlighted the role of *Gadd45b* in juvenile behavior and pro-inflammatory cytokine production^19^, synaptic plasticity, learning and memory and fear conditioning^20,21^, and psychosis in human brain^22^. Here, we provide results supporting the pro-depressant role of *Gadd45b* following CSDS. Interestingly, *Gadd45b* KD also reversed the social avoidance observed in our susceptible group following intra NAc viral infusions. The delayed effects of stress have been observed before in other mouse models^26–30^ and could be explained by the delayed induction of a stress phenotype exacerbated by an intermittent re-exposure to an aggressor or by surgeries and their recovery. While unexpected, these effects were still reversed by *Gadd45b KD* supporting its role on mediating susceptibility to social stress. Importantly, we also provide mechanistic insight into the epigenetic mechanisms by which *Gadd45b* might mediate its behavioral effects. Consistent with the findings presented here, previous studies reported changes in DNA methylation within the *Gad1* and *Ntrk2* gene promoters in rodent models of stress and humans suffering from schizophrenia, bipolar disorder, or depression^24,31,32^. *Dlx5* and *Nrtn* also came out as potential targets in DNA methylation and gene expression genome-wide profiling studies^6,10,12^. Altered methylation of these gene promoters could affect their transcriptional activity by regulating the binding of transcription factors and other transcriptional regulatory proteins. More work will be required to investigate these and other possible mechanisms downstream of the DNA methylation alterations found in our analyses.

The *Gadd45b* targets identified in this study include a GABAergic gene *(Gad1*), the BDNF TrkB receptor (*Ntrk2*), a gene important in VTA development (*Nrtn*), and *Dlx5* which is involved in several facets of brain development, each of which has been linked to stress phenotypes in previous investigations^24,31,32^. However, these findings remain preliminary, as global genome-wide assessment of DNA methylation and gene expression following *Gadd45b* KD will provide a more complete picture of the downstream targets of *Gadd45b* in the NAc in the context of stress susceptibility. In this sense, *DNMT3A* overexpression in the NAc, altering genome-wide epigenetic patterns, has previously been shown to increase susceptibility to CSDS and subchronic variable stress (CVS) in males, while its downregulation promotes resilience in females^10,12^. Consistently, loci specific^33^ and genome-wide reorganization^13,14,16^ of DNA methylation profiles has been described in the brain of suicide completers and in the blood of PTSD patients^17,34^. However, while DNA methylation is cell-type specific, it is likely that *Gadd45b* downstream targets might vary in a cell-type specific fashion as well. In the NAc, this would ultimately interfere with the activity of D1-type or D2-type medium spiny neuron populations differently as the two populations express distinct transcriptional profiles^35^ and are known to be affected by stress differently^4,36–38^. Future work should address these issues.

To conclude, our findings support the idea of a dynamic, activity-dependent process regulating behavioral susceptibility via changes in the epigenetic regulation of gene expression in the NAc of stressed mice. By being a highly plastic, but relatively stable epigenetic mark, DNA methylation exerts a dynamic control over gene expression in response to neuronal activity. While several processes are likely involved, our results suggest that *Gadd45b*, by being a downstream target of BDNF in the NAc, is involved in mediating these effects. Further explication of this novel pathway will contribute to improving our understanding of the molecular mechanisms underlying stress susceptibility.

## Methods

### Experimental subjects

Male 7–12-week-old C57BL/6 J mice (25–30 g, Jackson) and 4–6 month old CD1 retired breeders (35–45 g, Charles River) were used. Mice were fed *ad libitum* at 22~25 °C on a 12-hr light/dark cycle. CD1 mice were singly housed except during social defeats. All C57BL/6J mice were group housed before social defeats and singly housed after social defeats.

### Social defeat stress paradigm

CSDS was conducted as described previously^4,23^. Twenty-four hr after the last defeat, a social interaction test was performed. Based on social interaction ratios (time in interaction zone with social target/time in interaction zone without social target), mice were designated as susceptible or resilient: susceptible ratio <1; resilient ratio ≥1. For the effect of *Gadd45b* downregulation on social behaviors, HSV-*Gadd45b* miRNA (miR) or its control vector (HSV-GFP) were infused into the NAc 24 hr after the first social interaction test. Four days after the HSV infusion, the 2^nd^ social interaction test was performed. All experimental protocols were approved by the Institutional Animal Care and Use Committee (IACUC) at the Icahn School of Medicine at Mount Sinai (# LA12-00051) and at the Korea Brain Research Institute (# IACUC-15-00026). All experiments were performed in accordance with the guidelines of the IACUC at the Icahn School of medicine at Mount Sinai and at the Korea Brain Research Institute. All efforts were made to minimize animal suffering and to reduce the number of animals used.

### HSV vectors

Knockdown (KD) constructs designed to target *Gadd45b* mRNA were cloned using BLOCK-iT Pol II miR RNAi kit (Invitrogen). Briefly, four artificial miRNA oligonucleotides were designed using Invitrogen’s RNAi Designer (www.invitrogen.com/rnai) to KD *Gadd45b* and cloned into pcDNA6.2-GW. Mouse neuroblastoma (N2a) cells were transfected using lipofectamine 2000 (Invitrogen) with the different plasmids designed to target *Gadd45b* or LacZ as control. The level of *Gadd45b* mRNA KD was assessed using qRT-PCR 24 hr after transfection. The miRNA causing the most efficient downregulation was further Gateway cloned (Invitrogen) into the p1005 + HSV vector.

### Stereotaxic surgeries

Stereotaxic surgeries were performed as described previously^39^. Mice were anesthetized with a mixture of ketamine (100 mg/kg/10 ml) and xylazine (10 mg/kg/10 ml) (Henry Schein) in sterile saline. HSVs (-*Gadd45b* miR or -GFP) or BDNF (0.25 µg/side, recombinant human BDNF, R&D Systems) were bilaterally infused into the NAc (AP = 1.5, ML = ±1.5, and DV = −4.4 mm; 10° angle), while an AAV2 vector expressing ChR2, fused with enhanced yellow fluorescent protein (AAV2-EYFP-ChR2, purchased from University of North Carolina Vector Core) or its control (AAV2-EYFP), was infused into the VTA (AP = −3.2, ML = ±1.0, and DV = −4.6 mm; 7° angle). An infusion volume of 0.5 µl was delivered using 5 μL Hamilton syringe (Hamilton Company) over the course of 5 min (at a rate of 0.1 μl/min). Mice were allowed to recover for 4 days following the HSV infusion or for 7 days following the BDNF infusion before going through behavioral assessment. For the optogenetic stimulation of the VTA-NAc pathway, optic fibers were bilaterally implanted into the NAc (AP = 1.5; ML = ±1.3; DV = −3.9; 0° angle), three weeks after AAV2-EYFP-ChR2 or AAV2-EYFP infusion into VTA. Mice were allowed to recover for seven days following the cannulation, and then stimulated in home cages.

### Immunohistochemistry

Mice were anesthetized with a lethal dose of chloral hydrate and intracardially perfused with 0.1 M phosphate-buffered saline (PBS) and 4% (wt/vol) PBS-buffered paraformaldehyde 24 hr post social interaction test. Post-fixed brains were incubated overnight in 30% sucrose at room temperature before being sliced on a microtome at 40 μm on a microtome. Free-floating sections were washed with PBS and then blocked in 3% bovine serum albumin (BSA) and 0.3% Triton-X for 1 hr. For GFP labeling of localized HSV infusion, brain sections were incubated in 1:1000 of chicken anti-GFP (GFP-1020, Aves) in block solution overnight at 4 °C. The next day, sections were rinsed in PBS then incubated in 1:500 of donkey anti-chicken Cy2 (Immuno Research) in PBS for 1 hr then subsequently rinsed in PBS. All sections were counterstained and mounted with antifade solution, including DAPI then subsequently imaged on a LSM 710 confocal microscope.

### DNA methylation - MassARRAY EpiTYPER assays

Genomic DNA from the NAc was extracted using the triple prep kit from QIAgene according to the manufacturer’s instruction. DNA was sent to the Innovation Center of Genome Quebec where it was treated with sodium bisulfite (Na-BIS) using the Epitech Bisulfite kit (QIAgen) and where Epityper^40^ was performed.

### RNA Extraction and qRT-PCR

For RNA isolation, 14-gauge bilateral NAc punches were processed according to published protocol^39^. Primers were designed to amplify regions of 85–125 bp located within the target gene (Table 2). SYBR Green qRT-PCR was run in triplicate on the ABI7900HT Real-Time Cycler and analyzed using the ΔΔCt method as previously described^41^ with *Gapdh* as a normalization control. *Gapdh* mRNA expression was not altered among the groups in all experiments.

**Table 2:** Primers for real-time PCR.

Target gene		Primer sequence (5′–3′)
*Gapdh*	F	AACTTTGGCATTGTGGAAGG
	R	ACACATTGGGGGTAGGAACA
*Gadd45a*	F	TGAGCTGCTGCTACTGGAGA
	R	TCCCGGCAAAAACAAATAAG
*Gadd45b*	F	GCGGCCAAACTGATGAAT
	R	GATACCCGGACGATGTCAAT
*Gadd45g*	F	TCGCACAATGACTCTGGAAG
	R	GACTTTGGCGGACTCGTAGA
*Gad1*	F	GGCATCTTCCACTCCTTCGC
	R	ATCATACGTTGTAGGGCGCA
*Dlx5*	F	GCTACCCGGCCAAGGCTTAT
	R	CCATTCACCATCCTCACCTCTG
*Nrtn*	F	AGGAGGGTCTGCTCTTGGG
	R	AAAGTTCTCGAAGCTCCACCG
*Nrtk2*	F	ACTGTCCTGCTACCGCAGTT
	R	GGACTCTTTGGGTCGCAGAA

### Data analysis

Data were analyzed with Prism 6.0 (GraphPad) and SPSS. Phenotype and *Gadd45b* expression following CSDS was assessed using one-way ANOVA followed by Fisher’s LSD *post-hoc* tests. The effect of BDNF infusion and optogenetic stimulation on *Gadd45b* expression was assessed using independent sample t-test. The impact of *Gadd45b* KD on social interaction was tested using a mixed model two-way ANOVA with phenotype and viral infusion as main factors followed by Fisher’s PLSD *post-hoc* tests. MassARRAY EpiTYPER data were analyzed using mixed models ANOVAs with phenotype, viral infection and CpG as main factors followed by Fisher’s LSD *post-hoc* tests. Gene expression following *Gadd45b* KD was assessed using mixed model two-way ANOVAs with phenotype and viral infusion as main factors followed by Fisher’s LSD *post-hoc* tests. All *p* values of < 0.05 were considered to be statistically significant. All data are expressed as mean ± SEM.

### Ethics statement

All experimental protocols were approved by the Institutional Animal Care and Use Committee (IACUC) at the Icahn School of Medicine at Mount Sinai (# LA12-00051) and at the Korea Brain Research Institute (# IACUC-15-00026). All experiments were performed in accordance with the guidelines of the IACUC at the Icahn School of medicine at Mount Sinai and at the Korea Brain Research Institute. All efforts were made to minimize animal suffering and to reduce the number of animals used.

## Supplementary information

Supplementary information SREP-18-14402B
