# Supplementary material for: Gadd45b mediates depressive-like role through DNA demethylation
Source: Sci Rep. 2019 Mar 15;9:4615. doi: 10.1038/s41598-019-40844-8 (PMC6420662; doi:10.1038/s41598-019-40844-8)
Supplement: Supplementary file 1 — Supplementary information SREP-18-14402B [file 41598_2019_40844_MOESM1_ESM.pdf]

Gadd45b mediates depressive-like role through DNA demethylation

Benoit Labonté[1†], Yun Ha Jeong[2†], Eric Parise[3], Orna Issler[3], Mena Fatma[1], Olivia Engmann[3], Kyung-Ah Cho[2#], Rachael Neve[4], Eric J. Nestler[3], Ja Wook Koo[2*]

1. CERVO Brain Research Centre, Department of Neuroscience and Psychiatry, Faculty of Medicine, Laval University, Québec, Canada.
2. Department of Neural Development and Disease, Korea Brain Research Institute (KBRI), 61, Cheomdan-ro, Dong-gu, Daegu, 41068, Korea
3. Fishberg Department of Neuroscience, Friedman Brain Institute, Icahn School of Medicine at Mount Sinai, New York, NY 10029, USA.
4. Viral Gene Transfer Core, McGovern Institute for Brain Research, MIT, MA 02139, USA

[†] These authors contributed equally to this work

[#]Present address: Department of Molecular Medicine, Institute of Biotechnology, The University of Texas Health Science Center at San Antonio, San Antonio, TX 78229, USA

* To whom Correspondence should be addressed:

Ja Wook Koo, Department of Neural Development and Disease, Korea Brain Research Institute (KBRI), 61, Cheomdan-ro, Dong-gu, Daegu, 41068, Korea

Phone: 82-53-980-8430; email: jawook.koo@kbri.re.kr

Supplementary Figures

Supplementary Figure 1

Measurement of social interaction with the time spent in the interaction zone without a social target in each group and time in the interaction zone with a social target in each group **a,b**, Without target, mice in each group showed no difference in the time spent in the interaction zone.(**a**). However, with target, susceptible mice showed significantly reduced social interaction time compare with control and resilient group (**b**) [One-way analysis of variance (ANOVA), $F_{(2,27)}$=24.23, p<0.001, Control n=10, Susceptible n=10, Resilient n=10].

Supplementary Figure 2

mRNA expression levels of *Gadd45a* and *Gadd45g* in the NAc of mice that received intra-NAc infusion of HSV-GFP and HSV-Gadd45b miR. **a,b**, Both *Gadd45a* (**a**) and *Gadd45g* (**b**) mRNA levels in NAc are not different between HSV-GFP and HSV-Gadd45b miR group (**a**, $t_{(9)}$=0.0342, p=0.9735, HSV-GFP n=5, HSV-Gadd45b miR n=6, **b**, $t_{(9)}$=0.0134, p=0.9896, HSV-GFP n=5, HSV-Gadd45b miR n=6). mRNA expression levels were expressed as fold change (FC) compared to control group.

Supplementary Figure 3

Measurement of social interaction with the time spent in the interaction zone without a social target in each group and with a social target in each group at pre- and post-surgery of HSV-GFP or HSV-Gadd45b miR into NAc. **a,b**, Local *Gadd45b* KD in the NAc had no effect on social interaction in stress naïve animals [**a**, Mixed model two-way ANOVA, time effect: $F_{(1,32)}$=0.6393, p=0.4299; genetic effect: $F_{(1,32)}$=0.411, p=0.5260; time×genetic effect: $F_{(1,32)}$=0.0364, p=0.8499, HSV-GFP (pre- and post-) n=9, HSV-Gadd45b miR (pre- and post-) n=9, **b,** time effect: $F_{(1,32)}$=2.59, p=0.1173;

genetic effect: $F_{(1,32)}$=0.016, p=0.8995; time×genetic effect: $F_{(1,32)}$=0.0004, p=0.9841, HSV-GFP (pre- and post-) n=9, HSV-Gadd45b miR (pre- and post-) n=9]). **c,d**, in contrast, local *Gadd45b* KD in this brain region reverses the CSDS-induced social avoidance behavior in susceptible mice especially when the susceptible mice interact with social target [**c**, time effect: $F_{(1,44)}$=0.6767, p=0.4152; genetic effect: $F_{(1,44)}$=0.5857, p=0.4482; time×genetic effect: $F_{(1,44)}$=0.6631, p=0.4199, HSV-GFP (pre- and post-) n=11, HSV-Gadd45b miR (pre- and post-) n=13, **d,** time effect: $F_{(1,44)}$=1.429, p=0.2383; genetic effect: $F_{(1,44)}$=1.29, p=0.2623; time×genetic effect: $F_{(1,44)}$=6.923, p<0.05, HSV-GFP (pre- and post-) n=11, HSV-Gadd45b miR (pre- and post-) n=13]. **e,f**, after HSV-GFP infusion into NAc, resilient mice showed reduction of social interaction, whereas intra-NAc infusion of HSV-Gadd45b miR blocked this effect [**e**, time effect: $F_{(1,40)}$=1.387, p=0.2459; genetic effect: $F_{(1,40)}$=1.755, p=0.1928; time×genetic effect: $F_{(1,40)}$=0.5611, p=0.4582, HSV-GFP (pre- and post-) n=11, HSV-Gadd45b miR (pre- and post-) n=11, **f,** time effect: $F_{(1,40)}$=4.134, p<0.05; genetic effect: $F_{(1,40)}$=17.06, p<0.001; time×genetic effect: $F_{(1,40)}$=2.844, p=0.0995, HSV-GFP (pre- and post-) n=11, HSV-Gadd45b miR (pre- and post-) n=11]. Mixed model two-way ANOVA with Fisher's LSD *post-hoc* tests, *p < 0.05, **p < 0.01, ***p < 0.001. Bar graphs show mean ± SEM.

Supplementary Fig 1

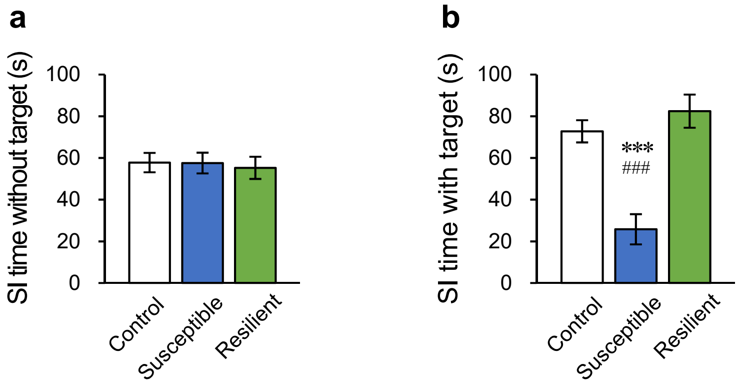

Supplementary Fig 2

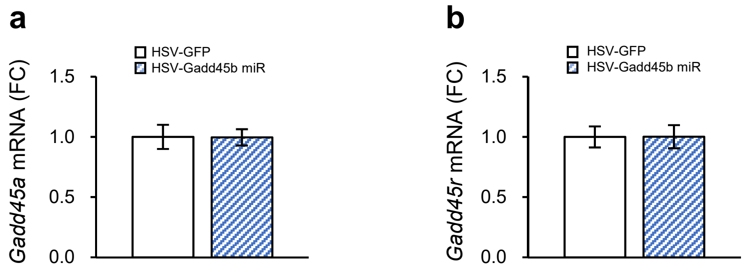

Supplementary Fig 3

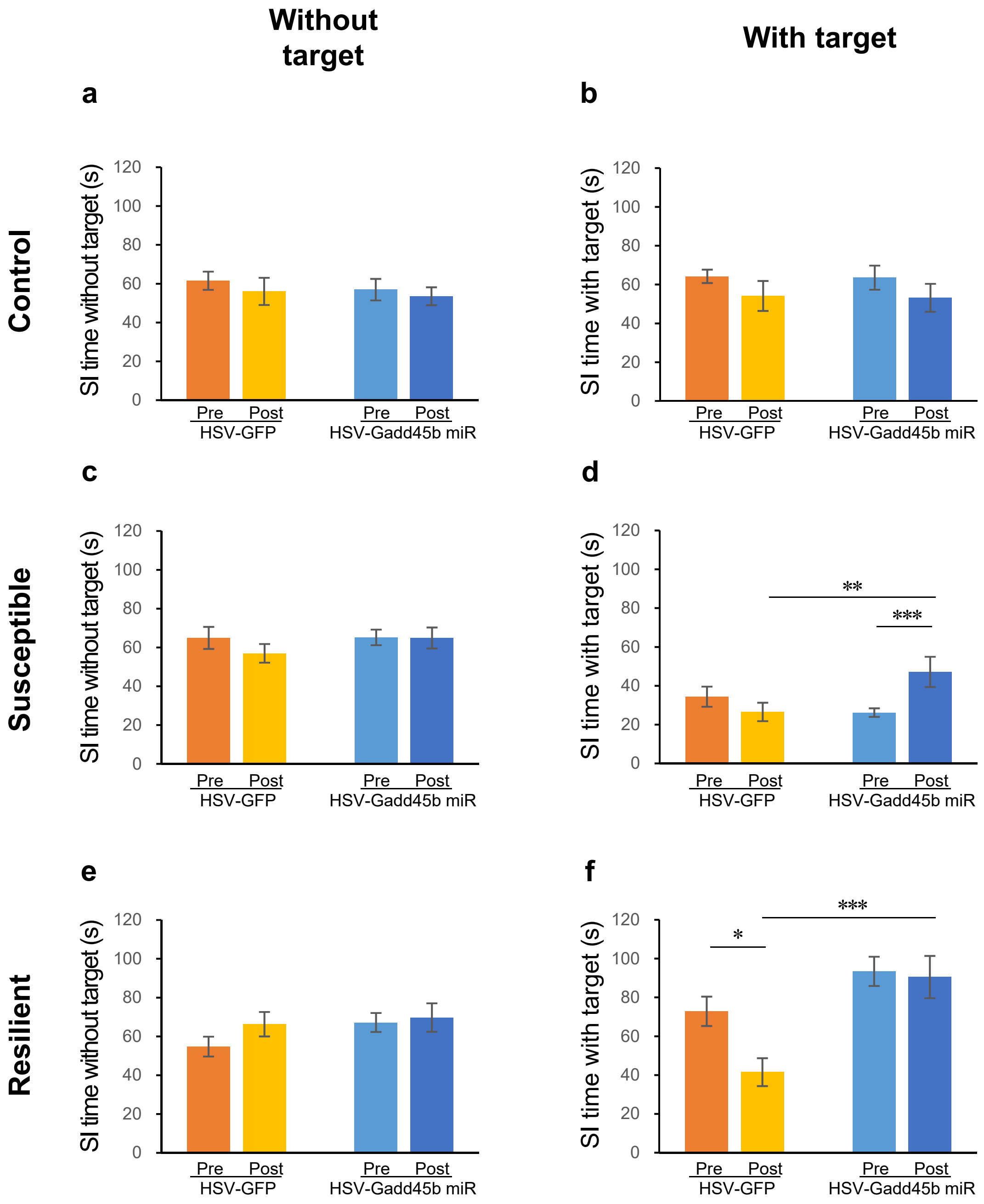

Supplementary Table 1. *P* values from post-hoc comparison between GFP vs. gadd45 KD in each group

a. DNA methylation at each CpG

Gad1

CpG	1			2,3			4,5			6,7			8			9			10,11			12.13			14			15			16-18			19			20			21			22			23		
Group	C	S	R	C	S	R	C	S	R	C	S	R	C	S	R	C	S	R	C	S	R	C	S	R	C	S	R	C	S	R	C	S	R	C	S	R	C	S	R	C	S	R	C	S	R	C	S	R
P value	ns	ns	ns	<0.001	<0.001	<0.001	ns	0.008	ns	ns	ns	ns	ns	ns	ns	ns	ns	ns	ns	0.028	ns	ns	ns	ns	ns	ns	ns	ns	ns	ns	ns	ns	ns	ns	ns	ns	ns	ns	ns	ns	ns	ns	ns	ns	ns	ns	ns	ns

Dlx5

CpG	1			2,3			4,5			6-8			9			10			11			12			13			14-17			18			19			20,21			22,23			24			25			26			27			28			29-31			32-34			35-38		
Group	C	S	R	C	S	R	C	S	R	C	S	R	C	S	R	C	S	R	C	S	R	C	S	R	C	S	R	C	S	R	C	S	R	C	S	R	C	S	R	C	S	R	C	S	R	C	S	R	C	S	R	C	S	R	C	S	R	C	S	R	C	S	R	C	S	R
P value	ns	0.026	ns	ns	ns	ns	ns	ns	ns	ns	ns	ns	ns	ns	ns	<0.001	0.001	ns	ns	ns	ns	ns	ns	ns	ns	ns	ns	ns	ns	ns	ns	ns	ns	ns	ns	ns	ns	ns	ns	ns	ns	ns	ns	ns	ns	ns	ns	ns	ns	ns	ns	ns	ns	ns	ns	0.023	ns	ns	ns	ns	ns	ns	ns	ns	ns	ns

Nrtn

CpG	1,2			3			4			5,6			7,8			9,10			11			12-14			29,30			31-33			34			35-36			37		
Group	C	S	R	C	S	R	C	S	R	C	S	R	C	S	R	C	S	R	C	S	R	C	S	R	C	S	R	C	S	R	C	S	R	C	S	R	C	S	R
P value	ns	ns	ns	ns	ns	ns	0.004	<0.001	0.003	ns	ns	ns	ns	ns	ns	ns	ns	ns	ns	ns	ns	ns	ns	ns	ns	ns	ns	ns	ns	ns	ns	ns	ns	ns	ns	ns	ns	ns	ns

Nrtk2

CpG	1			2-5			6,7			8			9			10			11			12			13,14			15			16,17			18			19-21			22,23			24			25			26			27			28			29			31			31,32			33,34		
Group	C	S	R	C	S	R	C	S	R	C	S	R	C	S	R	C	S	R	C	S	R	C	S	R	C	S	R	C	S	R	C	S	R	C	S	R	C	S	R	C	S	R	C	S	R	C	S	R	C	S	R	C	S	R	C	S	R	C	S	R	C	S	R	C	S	R	C	S	R
P value	ns	<0.001	ns	ns	ns	ns	ns	ns	ns	ns	ns	ns	ns	ns	ns	ns	ns	ns	ns	ns	ns	ns	ns	ns	ns	ns	ns	ns	ns	ns	ns	ns	ns	ns	ns	ns	ns	ns	ns	ns	ns	ns	ns	ns	ns	ns	ns	ns	ns	ns	ns	ns	ns	ns	<0.001	<0.001	<0.001	ns	ns	ns	ns	ns	ns	ns	ns	ns	ns	ns	ns

b. Total DNA methylation

	C	S	R
Gad1	0.021	0.001	ns
Dlx5	ns	<0.001	ns
Nrtn	ns	0.011	ns
Nrtk2	ns	ns	ns

c. mRNA expression

	C	S	R
Gad1	ns	ns	ns
Dlx5	0.02	0.002	0.007
Nrtn	ns	ns	ns
Nrtk2	0.04	0.004	0.01
